# Laboratory Work in Histology

**Published:** 1903-03

**Authors:** 


LABORATORY WORK IN HISTOLOGY.—By G. C. Hulen, M. D., Junior Professor of Anatomy and Director of the Histological Laboratory, University of Michigan. Third edition. Revised and enlarged. George Wahr, Publisher, Ann Arbor, Mich.

This work is in the nature of a guide for medical students. It is very complete in detail, and if followed with Dr. Huber through the university course of nine weeks, four hours a day, the student will be properly grounded in histology. T. J. B.

